# Unraveling the Association Between Cheese Consumption and Non‐Alcoholic Fatty Liver Disease: Insights From a Two‐Sample Mendelian Randomization Analysis

**DOI:** 10.1002/fsn3.70213

**Published:** 2025-05-10

**Authors:** Chen Ding, Shuwei Weng

**Affiliations:** ^1^ Department of Cardiology the First Affiliated Hospital of Fujian Medical University Fuzhou City Fujian Province China; ^2^ Clinical Research Center for Metabolic Heart Disease of Fujian Province Fuzhou City Fujian Province China; ^3^ Key Laboratory of Metabolic Heart Disease in Fujian Province Fuzhou City Fujian Province China; ^4^ Department of Cardiology The Fourth Affiliated Hospital of Soochow University, Suzhou Dushu Lake Hospital, Medical Center of Soochow University Suzhou City Jiangsu Province P. R. China; ^5^ Department of Cardiovascular Medicine The Second Xiangya Hospital, Central South University Changsha Hunan China; ^6^ Research Institute of Blood Lipid and Atherosclerosis Changsha Hunan China

**Keywords:** cheese consumption, dietary intervention, Mendelian randomization, non‐alcoholic fatty liver disease (NAFLD)

## Abstract

Non‐alcoholic fatty liver disease (NAFLD) is the most common chronic liver disease globally, and diet plays a crucial role in its progression. While dietary fats impact NAFLD, the specific effect of cheese consumption remains unclear. This study employs a two‐sample Mendelian randomization (MR) approach to explore the causal relationship between cheese intake and NAFLD, liver fat content, and liver fat proportion. Using summary‐level data from large genome‐wide association studies, we applied a two‐sample MR approach. Genetic variants linked to cheese consumption served as instrumental variables, selected under strict criteria, including genome‐wide significance and exclusion of pleiotropy. Robustness was ensured through various MR methods, including Inverse Variance Weighted (IVW) and MR‐Egger. MR analysis indicated that increased cheese consumption is negatively associated with NAFLD risk (OR = 0.589, 95% CI: 0.387–0.896, *p* = 0.014). This inverse relationship also extended to liver fat content (OR = 0.814, 95% CI: 0.689–0.960, *p* = 0.015) and liver fat proportion (OR = 0.830, 95% CI: 0.695–0.992, *p* = 0.04). No significant link was found between cheese intake and liver volume (OR = 0.976, 95% CI: 0.846–1.126, *p* = 0.737). Cheese intake may have a protective effect against NAFLD, potentially informing dietary management strategies. Further research is needed to confirm these findings across diverse populations.

## Graphical abstract

This study investigates the causal relationship between cheese consumption and non‐alcoholic fatty liver disease (NAFLD) using a two‐sample Mendelian randomization approach. The findings suggest that higher cheese intake may reduce the risk of NAFLD, offering potential insights for dietary management strategies.

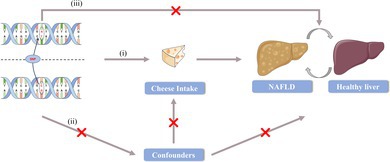

## 1 Introduction

Non‐alcoholic fatty liver disease (NAFLD) has become the most common chronic liver condition globally, closely linked to the prevalence of obesity and type 2 diabetes (Alharbi et al. 2014; Sun et al. 2022). Statistics indicate that the prevalence of NAFLD increased from approximately 25.26% between 1990 and 2006 to about 30.69% between 2016 and 2019, with significant variations due to regional and lifestyle factors (Younossi et al. 2023). NAFLD increases the risk of developing type 2 diabetes, cardiovascular diseases, and various cancers (Targher et al. 2021, 2020; Männistö et al. 2021; Tarantino et al. 2021). Its progression can lead to severe liver complications, including cirrhosis and hepatocellular carcinoma, highlighting the necessity of developing effective prevention and treatment strategies (Margini and Dufour 2016; Traussnigg et al. 2015).

Diet plays a crucial role in the development and management of NAFLD (Romero‐Gómez et al. 2017; Ahmed et al. 2024). Diets rich in saturated fats, sugars, and processed foods increase the risk and severity of NAFLD (Vancells Lujan et al. 2021), while diets high in omega‐3 fatty acids, fiber, and antioxidants can potentially mitigate this risk (Medina‐Urrutia et al. 2020; Fan and Cao 2013; Di Minno et al. 2012). However, the impact of specific foods, including cheese, on NAFLD is complex and not fully understood. Cheese is typically high in saturated fats but is also a source of essential nutrients like protein and calcium. It presents a paradox: while some observational studies suggest that cheese might have neutral or even beneficial effects on health due to its nutritional content (Astrup 2014; Azadbakht et al. 2005; Nilsen et al. 2015), other studies raise concerns about its potential to contribute to obesity and metabolic syndrome, which are closely linked to NAFLD (Yki‐Järvinen 2014; Godoy‐Matos et al. 2020).

In this study, we employed a two‐sample Mendelian randomization approach to not only reveal the causal relationship between cheese intake and NAFLD but also to explore the association between cheese intake and several variables closely related to the progression of NAFLD, such as liver fat content, liver volume, and percent liver fat. By using Mendelian randomization, we were able to overcome typical confounding factors encountered in dietary studies, providing robust genetic evidence for dietary recommendations for NAFLD patients.

## 2 Methods

### 2.1 Mendelian Randomization

**FIGURE 1:**
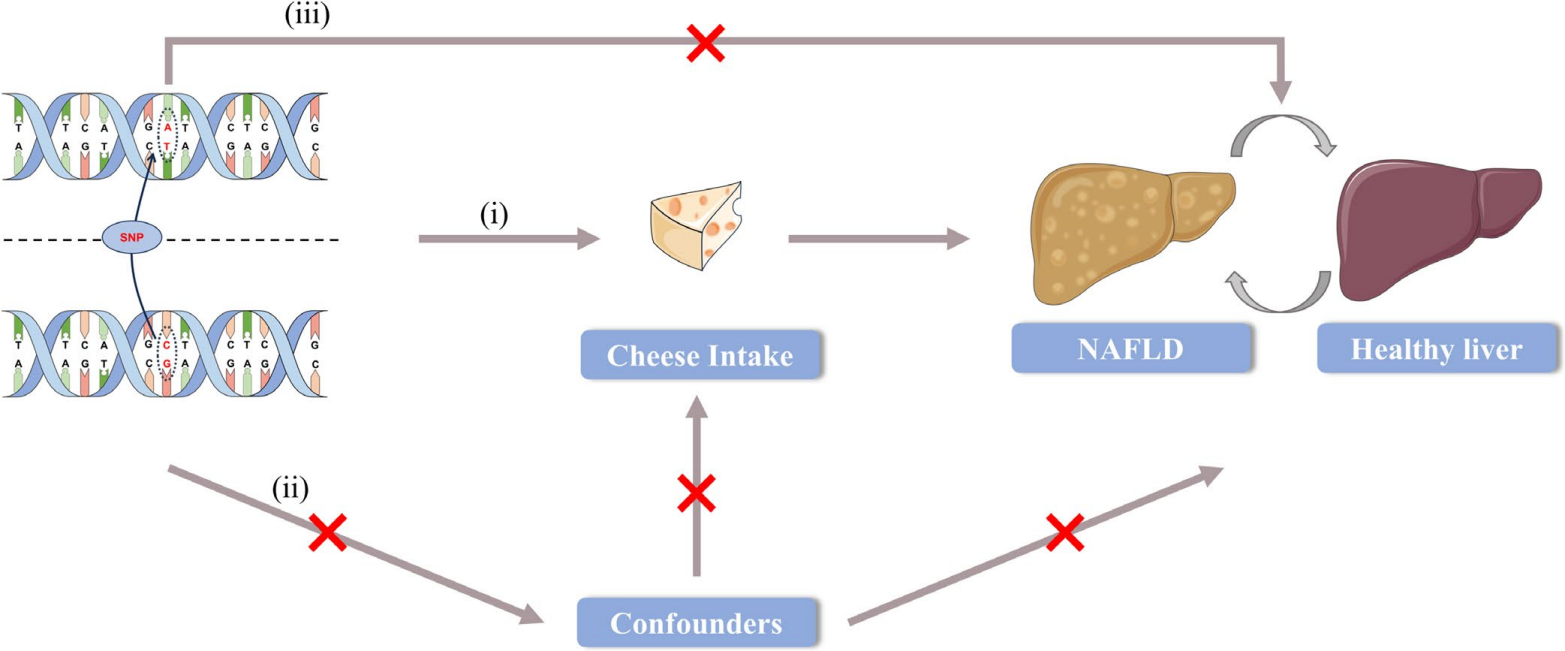
Plot of three key assumptions.

In this study, a two‐sample Mendelian randomization (MR) design based on summary‐level data was employed to investigate the causal relationship between variables. The MR analysis relies on the following assumptions regarding genetic variants: (i) they are strongly associated with the exposure of interest (relevance assumption), (ii) they are independent of confounding variables that may affect the exposure‐outcome relationship (independence assumption), and (iii) they influence the outcome solely through the exposure and not via any other causal pathways (exclusion restriction assumption) (Figure 1) (Skrivankova et al. 2021). Ethical approval was not required for this study as it utilized summary statistics and did not involve human subjects.

### 2.2 Selection of Exposure and Outcome Variables

We compiled essential characteristics of the exposure/outcome data, comprising GWAS‐ID, PMID, first author, year, population details, gender distribution, sample size, variant count, and consortium information (Table 1). The MR analysis was particularly oriented towards data from European populations, ensuring no overlap among the cohorts involved.

**TABLE 1:** Characteristics of data of cheese intake and NAFLD.

	Exposures/Outcomes	GWAS‐ID	Details
Exposure	Cheese intake	ukb‐b‐1489	PMID: NA
			Year: 2018
			Author: Ben Elsworth
			Population: European
			Sex: Males and Females
			Sample size: 451486
			Number of SNPs: 8851867
			Consrtium: MRC‐IEU
Outcome	NAFLD	ebi‐a‐GCST90054782	PMID: 34535985
			Year: 2021
			Author: Fairfield Cj
			Population: European
			Sex: NA
			Sample size: 377998
			Number of SNPs: 9097254
			Consrtium: NA
	Liver fat	ebi‐a‐GCST90029073	PMID: 34957434
			Year: 2021
			Author: Haas ME
			Population: European
			Sex: NA
			Sample size: 32974
			Number of SNPs: 9499333
			Consrtium: NA
	Liver volume	ebi‐a‐GCST90016666	PMID: 34128465
			Year: 2021
			Author: Liu Y
			Population: European
			Sex: NA
			Sample size: 32860
			Number of SNPs: 9275407
			Consrtium: NA
	Percent liver fat	ebi‐a‐GCST90016673	PMID: 34128465
			Year: 2021
			Author: Liu Y
			Population: European
			Sex: NA
			Sample size: 32858
			Number of SNPs: 9275407
			Consrtium: NA

### 2.3 Selection and Validation of SNPs

In the selection and validation of SNPs, we adhered to the following criteria: Firstly, SNPs associated with cheese intake were selected based on a genome‐wide significance threshold of *p* < 5 × 10^−8^. Secondly, the independence of the selected SNPs was assessed through linkage disequilibrium checks, excluding those with an *r* ^2^ > 0.001. Thirdly, SNPs with an F‐statistic greater than 10 were identified and filtered to ensure robustness. Furthermore, SNPs potentially related to confounding factors were excluded using LDlink (https://ldlink.nih.gov/?tab=ldtrait), aiming to minimize the interference caused by confounders (Tables S1 and S2).

### 2.4 Mendelian Randomization Analysis

In our study, we employed ten different methods for Mendelian randomization analysis, selecting the multiplicative random effects Inverse Variance Weighted (IVW) approach as the primary MR analysis due to its optimal accuracy and superior instrumental variable (IV) quality. The remaining methods, including Maximum Likelihood, Inverse Variance Weighted (Fixed Effects), Simple Mode, Weighted Median, Weighted Mode, MR Egger, MR‐PRESSO, and MR RAPS, served as complementary approaches to assess the causal relationship between exposure and outcome.

### 2.5 Sensitivity Analysis and Pleiotropy Analysis

We employed MR‐Egger and IVW methods to perform Cochran's Q test to assess heterogeneity among the individual instrumental variables. The MR‐Egger intercept test was used to evaluate pleiotropy, with a significant *p*‐value of the MR‐Egger intercept indicating the presence of pleiotropy. The MR‐PRESSO method serves as a robust complement in testing for pleiotropy within MR analysis. Additionally, we conducted leave‐one‐out analysis to identify potential influential SNPs that significantly impact the residual IVW results (Figure S1). Through the Visual SNPs Plot (Figure S2), we can determine the association between each SNP site and the outcome of NAFLD. The funnel plot (Figure S3) was utilized to visualize potential heterogeneity and pleiotropy in Mendelian randomization analysis.

### 2.6 Statistical Analysis

All analyses were conducted using R software (version 4.1.2), with the Mendelian randomization analysis component utilizing the “TwoSampleMR” package (version 0.5.11). The “MRPRESSO” package (version 1.0) was employed for the MR‐PRESSO method, and the “mr.raps” package (version 0.2) was used for the RAPS method. In all primary analyses, as well as in pleiotropy and sensitivity analyses, a *p*‐value of less than 0.05 was considered statistically significant.

## 3 Results

### 3.1 Assessment of Mendelian Randomization Analysis Results

**FIGURE 2:**
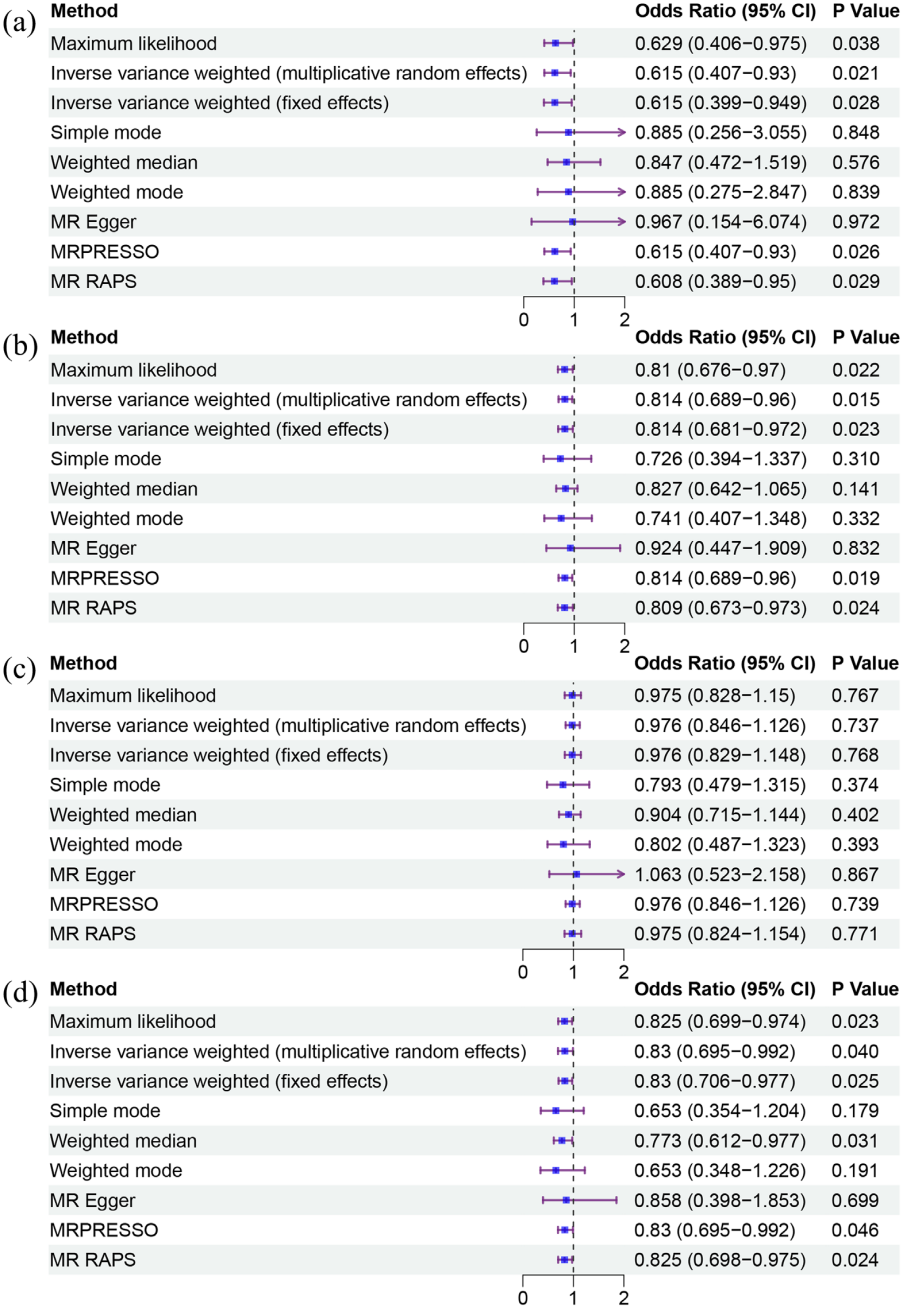
The forest plot of the causal effect of cheese intake on different outcome variables. (a) NAFLD; (b) liver fat; (c) liver volume; (d) percent liver fat.

**FIGURE 3:**
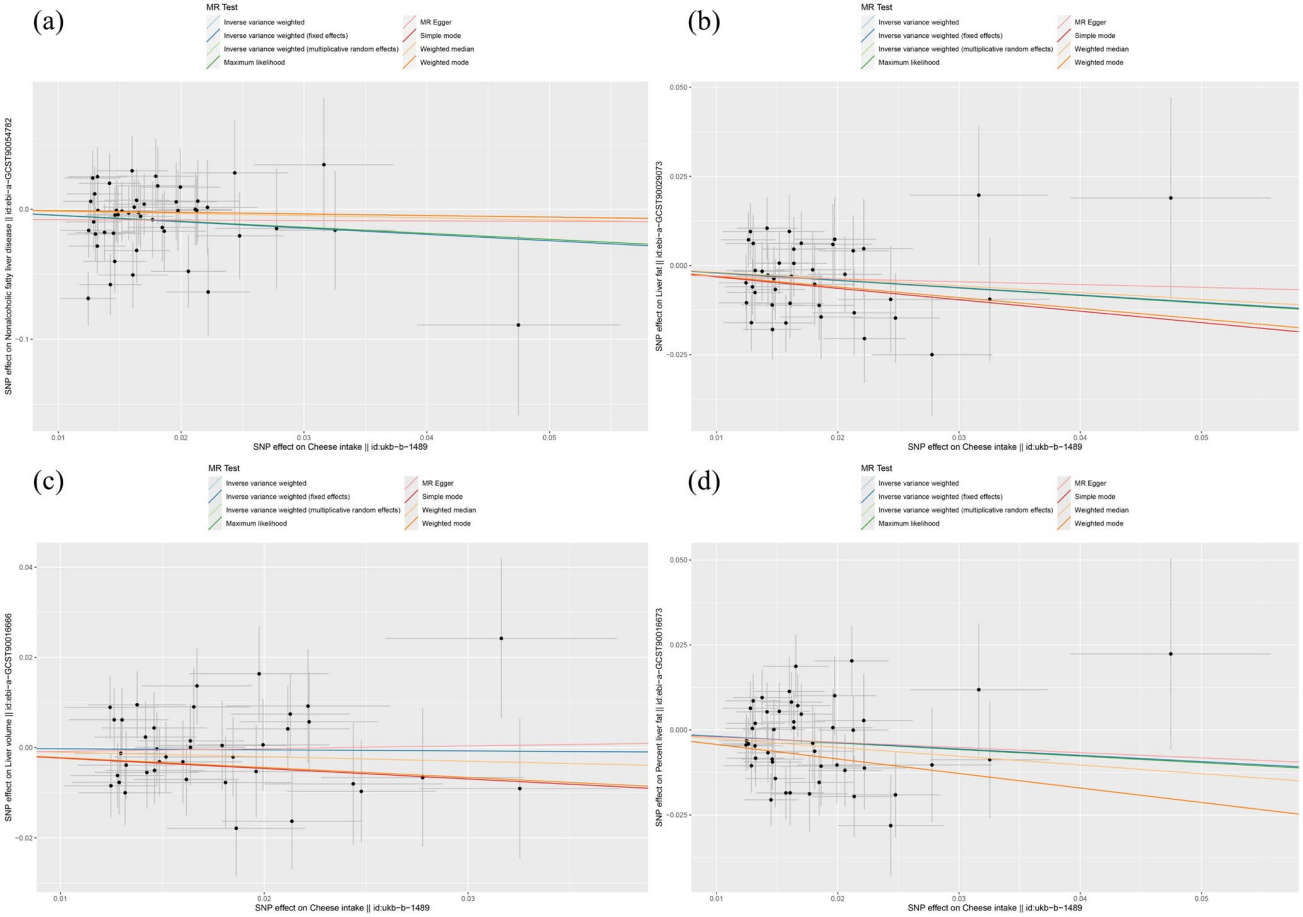
Scatter plot of MR analysis using different MR methods. (a) NAFLD; (b) liver fat; (c) liver volume; (d) percent liver fat.

Based on the results from the multiplicative random effects IVW method, genetically predicted cheese consumption increase by one standard deviation is negatively associated with the risk of NAFLD (OR = 0.589, 95% CI: 0.387–0.896, *p* = 0.014). This association was also observed using the Maximum Likelihood, Fixed Effects IVW, MR‐PRESSO, and MR RAPS methods. Additionally, cheese intake was significantly negatively associated with liver fat and percent liver fat (OR = 0.814, 95% CI: 0.689–0.960, *p* = 0.015 and OR = 0.830, 95% CI: 0.695–0.992, *p* = 0.04, respectively). However, cheese intake did not appear to be significantly associated with liver volume (OR = 0.976, 95% CI: 0.846–1.126, *p* = 0.737) (Figure 2 and Table S3). The negative correlations between cheese intake and NAFLD, liver fat, and percent liver fat are clearly depicted in the scatter plots (Figure 3). It is important to note that the methods other than multiplicative random effects IVW have limitations in terms of statistical assumptions and precision. Therefore, the lack of statistical significance in some methods does not imply the absence of a correlation between the exposure and outcome variables. The significant associations observed serve only as indicative evidence. Our Mendelian randomization analysis is primarily based on the multiplicative random effects IVW results, with other methods providing supportive evidence.

### 3.2 Sensitivity Analysis and Pleiotropy Assessment

In the summary, Cochran's Q test conducted using the MR‐Egger and IVW methods, we did not detect significant heterogeneity between cheese intake and NAFLD, liver fat, liver volume, or percent liver fat (Table 2). The symmetrical distribution of scatter points in the funnel plots further supports the conclusion of no significant heterogeneity (Figure S3). Although the leave‐one‐out analysis indicated that the removal of certain SNPs might affect the significance of the results, suggesting potential pleiotropy (Figure S1), the MR‐Egger intercept test provided no evidence of pleiotropy between cheese intake and NAFLD, liver fat, liver volume, or percent liver fat. This finding is consistent with the results of the MR‐PRESSO global test (Table 2).

**TABLE 2:** Pleiotropy and heterogeneity tests.

	Test	Method	Results
NAFLD	Pleiotropy	MRPRESSO	RSSobs (global test): 44.43693
			*p*‐value (global test): 0.685
		MR‐Egger intercept test	Estimated value of the intercept: −0.007715641
			Standard error: 0.01553657
			*p*‐value: 0.6218281
	Heterogeneity	MR‐Egger	Cochran's Q: 42.50222
			Degree of freedom: 46
			*p*‐value: 0.6195580
		IVW	Cochran's Q: 42.74884
			Degree of freedom: 47
			*p*‐value: 0.6491687
Liver fat	Pleiotropy	MRPRESSO	RSSobs (global test): 36.55904
			*p*‐value (global test): 0.711
		MR‐Egger intercept test	Estimated value of the intercept: −0.002169666
			Standard error: 0.00612106
			*p*‐value: 0.7249045
	Heterogeneity	MR‐Egger	Cochran's Q: 34.67449
			Degree of freedom: 39
			*p*‐value: 0.6674379
		IVW	Cochran's Q: 62.71730
			Degree of freedom: 40
			*p*‐value: 0.7030511
Liver volume	Pleiotropy	MRPRESSO	RSSobs (global test): 31.62761
			*p*‐value (global test): 0.836
		MR‐Egger intercept test	Estimated value of the intercept: −0.0014432
			Standard error: 0.005954443
			*p*‐value: 0.8097941
	Heterogeneity	MR‐Egger	Cochran's Q: 30.03942
			Degree of freedom: 38
			*p*‐value: 0.8180772
		IVW	Cochran's Q: 30.09816
			Degree of freedom: 39
			*p*‐value: 0.8460604
Percent liver fat	Pleiotropy	MRPRESSO	RSSobs (global test): 58.94442
			*p*‐value (global test): 0.166
		MR‐Egger intercept test	Estimated value of the intercept: −0.0005685418
			Standard error: 0.006500844
			*p*‐value: 0.9306882
	Heterogeneity	MR‐Egger	Cochran's Q: 56.35550
			Degree of freedom: 46
			*p*‐value: 0.1409198
		IVW	Cochran's Q: 56.36487
			Degree of freedom: 47
			*p*‐value: 0.1645133

Abbreviation: RSSobs, observed residual sum of squares.

## 4 Discussion

In this two‐sample Mendelian randomization study, based on large‐scale GWAS summary statistics and results calculated using the IVW method with a random effects model, we found that cheese intake is negatively associated with the occurrence of NAFLD, liver fat, and percent liver fat.

Cheese, as a versatile dairy product, often serves as an important source of protein and fat in the Mediterranean diet. Most existing research on cheese and NAFLD has been observational clinical studies. For instance, the FASA Persian cohort study (Keshavarz et al. 2022) found that subjects with a higher fatty liver index had higher dairy consumption compared to those with a lower index, but there was no significant association between cheese intake and the fatty liver index. A large‐scale prospective cohort study in a Korean population (Lee et al. 2021) showed that, in men and women over the age of 50, the highest tertile of dairy protein intake was significantly associated with a reduced incidence of NAFLD compared to the lowest tertile. However, the reduction in NAFLD incidence related to cheese intake was only significant in women over 50. A previous meta‐analysis (Yuzbashian et al. 2023) indicated that dairy consumption generally shows a moderate but consistent negative correlation with the occurrence of NAFLD. Specifically, consumption of milk and yogurt was slightly associated with a decrease in NAFLD incidence, while cheese intake did not show a significant association with the occurrence of NAFLD, and there was moderate heterogeneity. Unlike NAFLD, clinical studies on the relationship between cheese intake and liver fat content are still lacking. This may be due to the high cost of optimal methods for measuring liver fat content, such as MRI and MRS, which makes it difficult to implement in large‐scale studies. Consequently, sample sizes are often small, making it challenging to obtain statistically significant results.

Although our analysis, using genetic variants as instrumental variables, does not directly explain the mechanism, existing literature provides some possible biological explanations. Research by the Higurashi team (Higurashi et al. 2016) suggests that cheese intake can significantly reduce the accumulation of triglycerides and cholesterol in the liver, thereby lowering the risk of metabolic syndrome. Studies by Murru et al. (2018) further confirm that cheese rich in conjugated linoleic acid can enhance plasma DHA levels by increasing PPAR‐α activity, aiding in normal lipid metabolism. Moreover, the negative regulation of NAFLD by cheese likely involves its unique anti‐inflammatory properties. Kadooka et al. (Hosoya et al. 2012) found an increase in Treg cell expression in aggregated lymphoid nodules in mice following a cheese‐inclusive diet intervention and observed a significant reduction in inflammatory factors IL‐4, IL‐10, and IL‐17. Additionally, Lordan and Zabetakis (2017) in his review highlighted that the fat component of dairy products, particularly in cheese, shows potential bioactivity against chronic inflammation. He emphasized that different types of fatty acids and bioactive lipids in cheese might alleviate inflammation by modulating inflammatory pathways, such as inhibiting the NF‐κB pathway. These foundational research findings support the view that there is a negative correlation between cheese intake and NAFLD and reveal the potential mechanisms by which cheese may exert its protective effects through the regulation of lipid metabolism and inflammatory responses.

Indeed, this article still has certain limitations. First, the data included in this article are all from European populations, and the genetic variants used may have different expressions in different populations or subgroups, which could limit the external generalizability of the results. Secondly, although Mendelian randomization overcomes the effects of confounding factors at the genetic level, the potential interactions between genetic variants and environmental factors cannot be completely ruled out. Finally, although Mendelian randomization provides strong evidence of causality, interpreting this relationship from a mechanistic perspective remains challenging.

## 5 Conclusion

In summary, this study reveals a negative causal relationship between cheese intake and NAFLD, as well as liver fat content and percent liver fat. This finding provides new scientific evidence for the potential role of cheese in metabolic regulation and may offer new dietary management strategies for patients with NAFLD. Therefore, our research not only expands our understanding of the relationship between cheese and liver health but also lays the foundation for further nutritional intervention studies.

## Author Contributions

**Chen Ding:** data curation (lead), writing – original draft (lead). **Shuwei Weng:** conceptualization (lead), formal analysis (lead), investigation (lead), methodology (lead), project administration (lead), resources (lead), software (lead), supervision (lead), validation (lead), visualization (lead), writing – review and editing (lead).

## Conflicts of Interest

The authors declare no conflicts of interest.

## Supporting information


Data S1.
**Figure S1.** Leave‐one‐out plots.
**Figure S2.** Visual SNPs plots.
**Figure S3.** Funnel plots.
**Table S1.** Characteristics of instrumental variables.
**Table S2.** The LDlink results of the instrumental variables used in this.
**Table S3.** Feature variable selection table.

## Data Availability

All the raw data in the article come from the GWAS database, and the analysis results are presented in the main text and Supporting Information.
