# Supplementary material for: Unraveling the Association Between Cheese Consumption and Non‐Alcoholic Fatty Liver Disease: Insights From a Two‐Sample Mendelian Randomization Analysis
Source: Food Sci Nutr. 2025 May 10;13(5):e70213. doi: 10.1002/fsn3.70213 (PMC12064983; doi:10.1002/fsn3.70213)
Supplement: Supplementary file 1 — Data S1. Figure S1. Leave‐one‐out plots. Figure S2. Visual SNPs plots. Figure S3. Funnel plots. Table S1. Characteristics of instrumental variables. Table S2. The LDlink results of the instrumental variables used in this. Table S3. Feature variable selection table. [file FSN3-13-e70213-s001.pdf]

Supplementary Material

1 Supplementary Figures

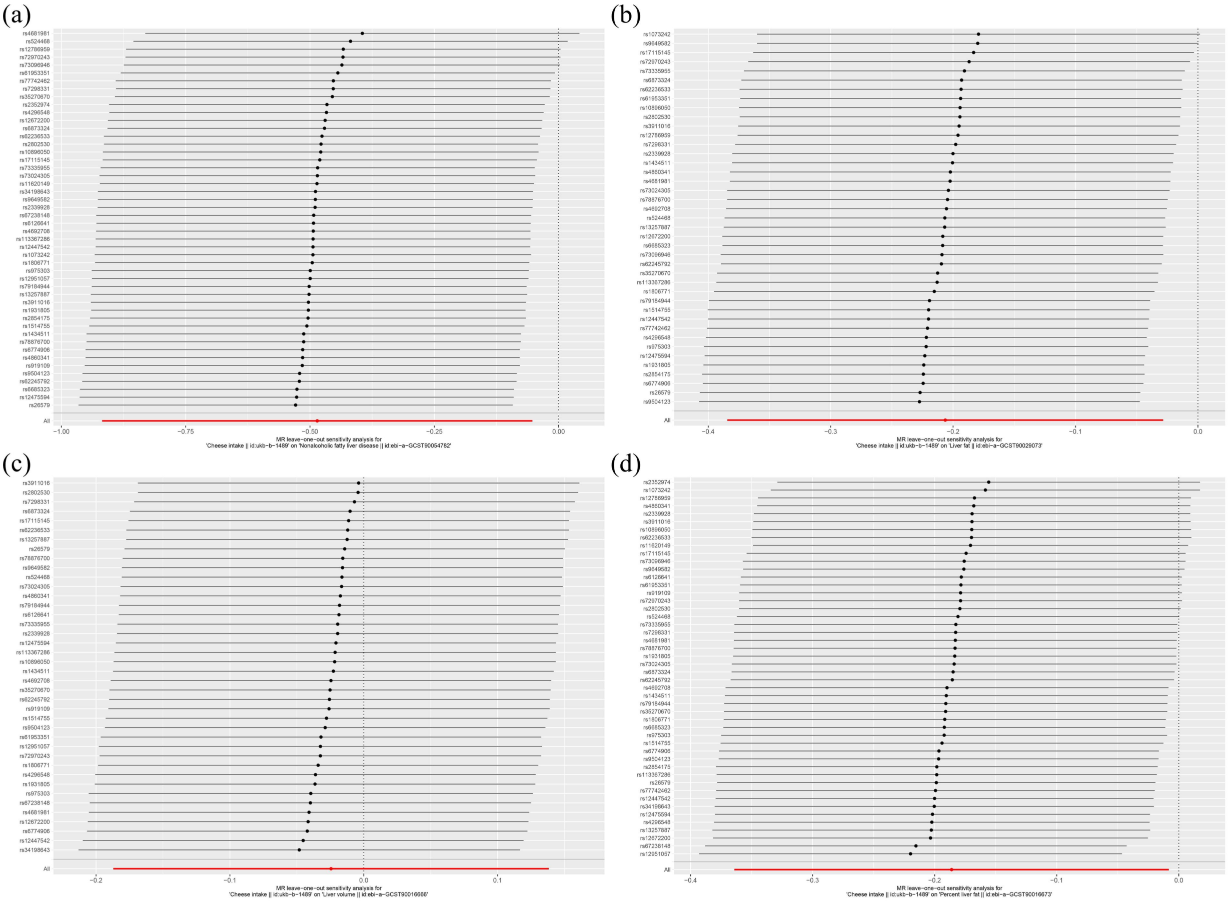

Supplementary Figure 1. Leave-one-out plots.

(a) NAFLD; (b) Liver fat; (c) Liver volume; (d) Percent liver fat

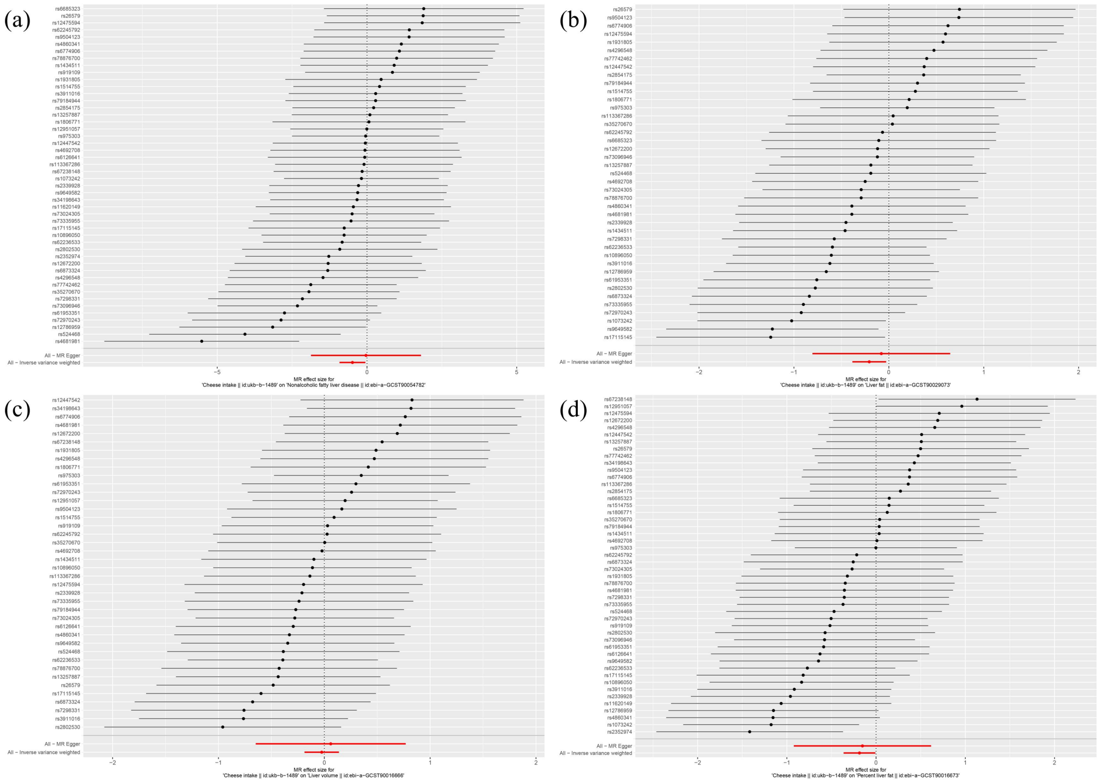

Supplementary Figure 2. Visual SNPs plots.

(a) NAFLD; (b) Liver fat; (c) Liver volume; (d) Percent liver fat

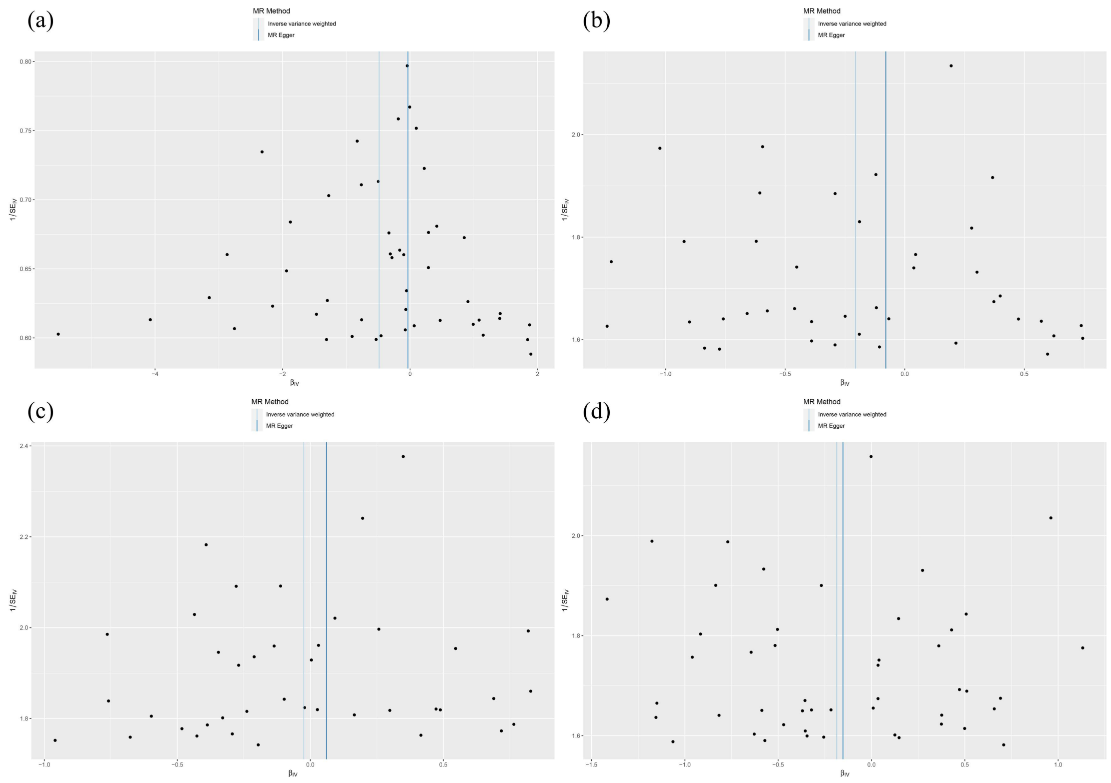

Supplementary Figure 3. Funnel plots.

(a) NAFLD; (b) Liver fat; (c) Liver volume; (d) Percent liver fat

2 Supplementary Tables

（Supplementary Table 1 and 2 are too large to be displayed completely in this document. For details, please refer to the editable CSV files uploaded.）

Supplementary Table 1. Characteristics of instrumental variables.

Supplementary Table 2. The LDlink results of the instrumental variables used in this study.

Exposures	Method	Number of SNPs	Beta	Standard Error	Odds Ratio (95% CI)	P Value
NAFLD	Maximum likelihood	48	-0.463967	0.223657	0.629 (0.406-0.975)	**0.008**
	Inverse variance weighted (multiplicative random effects)		-0.485429	0.210499	0.615 (0.407-0.930)	**0.014**
	Inverse variance weighted (fixed effects)		-0.485429	0.220718	0.615 (0.399-0.949)	**0.008**
	Simple mode		-0.122021	0.631962	0.885 (0.256-3.055)	0.886
	Weighted median		-0.166575	0.298209	0.847 (0.472-1.519)	0.576
	Weighted mode		-0.122021	0.596092	0.885 (0.275-2.847)	0.889
	MR Egger		-0.033049	0.937294	0.967 (0.154-6.074)	0.873
	MRPRESSO		-0.485430	0.210500	0.615 (0.407-0.930)	**0.026**
	MR RAPS		-0.497686	0.227794	0.608 (0.389-0.950)	**0.029**
Liver fat	Maximum likelihood	41	-0.210748	0.092076	0.81 (0.676-0.970)	**0.022**
	Inverse variance weighted (multiplicative random effects)		-0.206379	0.084711	0.814 (0.689-0.960)	**0.015**
	Inverse variance weighted (fixed effects)		-0.206379	0.090819	0.814 (0.681-0.972)	**0.023**
	Simple mode		-0.320248	0.311756	0.726 (0.394-1.337)	0.31
	Weighted median		-0.189863	0.128907	0.827 (0.642-1.065)	**0.141**
	Weighted mode		-0.300286	0.305748	0.741 (0.407-1.348)	0.332
	MR Egger		-0.079141	0.370274	0.924 (0.447-1.909)	0.832
	MRPRESSO		-0.206379	0.084711	0.814 (0.689-0.960)	**0.019**
	MR RAPS		-0.211415	0.093847	0.809 (0.673-0.973)	**0.024**
Liver volume	Maximum likelihood	40	-0.024853	0.083893	0.975 (0.828-1.150)	0.767
	Inverse variance weighted (multiplicative random effects)		-0.024464	0.072902	0.976 (0.846-1.126)	0.737
	Inverse variance weighted (fixed effects)		-0.024464	0.082985	0.976 (0.829-1.148)	0.768
	Simple mode		-0.231707	0.257831	0.793 (0.479-1.315)	0.374
	Weighted median		-0.100479	0.119945	0.904 (0.715-1.144)	0.402
	Weighted mode		-0.220041	0.254964	0.802 (0.487-1.323)	0.393
	MR Egger		0.060804	0.361460	1.063 (0.523-2.158)	0.867
	MRPRESSO		-0.024464	0.072902	0.976 (0.846-1.126)	0.739
	MR RAPS		-0.024995	0.085719	0.975 (0.824-1.154)	0.771
Percent liver fat	Maximum likelihood	48	-0.192275	0.084439	0.825 (0.699-0.974)	**0.023**
	Inverse variance weighted (multiplicative random effects)		-0.186135	0.090812	0.830 (0.695-0.992)	**0.04**
	Inverse variance weighted (fixed effects)		-0.186135	0.082925	0.830 (0.706-0.977)	**0.025**
	Simple mode		-0.426574	0.312518	0.653 (0.354-1.204)	0.179
	Weighted median		-0.257410	0.119576	0.773 (0.612-0.977)	**0.031**
	Weighted mode		-0.426574	0.321573	0.653 (0.348-1.226)	0.191
	MR Egger		-0.152748	0.392633	0.858 (0.398-1.853)	0.699
	MRPRESSO		-0.186135	0.090812	0.830 (0.695-0.992)	**0.046**
	MR RAPS		-0.192440	0.085227	0.825 (0.698-0.975)	**0.024**

Supplementary Table 3. Feature Variable Selection Table.
